# Harnessing the potential of bulk segregant analysis sequencing and its related approaches in crop breeding

**DOI:** 10.3389/fgene.2022.944501

**Published:** 2022-08-08

**Authors:** Aasim Majeed, Prerna Johar, Aamir Raina, R. K. Salgotra, Xianzhong Feng, Javaid Akhter Bhat

**Affiliations:** ^1^ School of Agricultural Biotechnology, Punjab Agriculture University (PAU), Ludhiana, India; ^2^ School of Biotechnology, Sher-e-Kashmir University of Agricultural Sciences and Technology of Jammu, Jammu, India; ^3^ Department of Botany, Faculty of Life Sciences, Aligarh Muslim University, Aligarh, India; ^4^ Zhejiang Lab, Hangzhou, China; ^5^ International Genome Center, Jiangsu University, Zhenjiang, China

**Keywords:** QTL-seq, MutMap, next-generation sequencing, crop breeding, fine-mapping

## Abstract

Most plant traits are governed by polygenes including both major and minor genes. Linkage mapping and positional cloning have contributed greatly to mapping genomic loci controlling important traits in crop species. However, they are low-throughput, time-consuming, and have low resolution due to which their efficiency in crop breeding is reduced. In this regard, the bulk segregant analysis sequencing (BSA-seq) and its related approaches, viz., quantitative trait locus (QTL)-seq, bulk segregant RNA-Seq (BSR)-seq, and MutMap, have emerged as efficient methods to identify the genomic loci/QTLs controlling specific traits at high resolution, accuracy, reduced time span, and in a high-throughput manner. These approaches combine BSA with next-generation sequencing (NGS) and enable the rapid identification of genetic loci for qualitative and quantitative assessments. Many previous studies have shown the successful identification of the genetic loci for different plant traits using BSA-seq and its related approaches, as discussed in the text with details. However, the efficiency and accuracy of the BSA-seq depend upon factors like sequencing depth and coverage, which enhance the sequencing cost. Recently, the rapid reduction in the cost of NGS together with the expected cost reduction of third-generation sequencing in the future has further increased the accuracy and commercial applicability of these approaches in crop improvement programs. This review article provides an overview of BSA-seq and its related approaches in crop breeding together with their merits and challenges in trait mapping.

## Introduction

Identification and dissection of genetic loci determining a particular trait is a regular process in genetics. Most of the complex quantitative traits are regulated by multiple loci distributed across the genome of a species. So to precisely detect the specific genetic elements linked with the trait of interest, one has to link all the loci with that trait (Bhat and Yu, 2021). A quantitative trait locus (QTL) is defined as a region within the genome that is associated with the genetic variation of a quantitative trait. QTL mapping is a widely accepted and applied approach to identify the genes/QTLs determining a complex quantitative trait. Moreover, positional cloning and QTL mapping are the two powerful approaches to dissect the genetic basis of phenotypic variation of important agronomic traits. Both these approaches investigate the genomes for polymorphic markers, followed by linking the polymorphic markers with a particular trait to identify the most likely candidate genomic regions controlling that trait. At the next level, increasing the marker density across these candidate regions would ensure further refinement of their physical interval (fine-mapping), followed by the evaluation of their actual physical position on the chromosomes (physical mapping). Diverse QTLs and underlying genes for numerous traits across a myriad of species have been successfully deciphered using these approaches. The major limitation to these approaches, however, is that they usually are low-throughput and time-consuming (Song et al., 2017).

The bulk segregant analysis is a high-throughput QTL mapping approach to rapidly identify genomic loci regulating the trait of interest. In contrast to individual segregant analysis (ISA), which classifies segregants according to their marker genotypes, the BSA pools segregants according to their phenotypes. When the former compares trait values of different classes, the latter compares marker allele frequencies in different classes (Huang et al., 2020). Although ISA is more commonly used, however, due to more precision and power of BSA and its simplicity, quickness, and cheaper nature than ISA, it provides additional advantages as compared to ISA. The brisk evolution of sequencing technologies along with the rapid downfall of sequencing costs has put the BSA approach to a newer level by integrating the traditional BSA approach with NGS. The basis of BSA is to generate two phenotypically contrasting groups or populations by crossing two extreme phenotypes. This is followed by creating two bulks from the segregating populations, i.e., F_2_ by selecting individuals with contrasting phenotypes; for example, tall and short plants, tolerant and susceptible plants, etc. (Zhang and Panthee, 2020). The key to this approach is that the alleles of a locus controlling the trait would be enriched in either bulk; for example, the allele “A” can occur frequently in the tolerant plants, and the allele “a” frequently exists in the susceptible plants, whereas those not affecting the trait would segregate randomly in both bulks (Zhang and Panthee, 2020). BSA was initially targeted to develop genetic markers for trait dissection at earlier stages (Giovannoni et al., 1991; Michelmore et al., 1991). Both marker development and genetic mapping were time-consuming and labor-intensive. However, the rapid advancement of sequencing technologies has greatly facilitated marker discovery and their associations with traits of interest. Integrating BSA with sequencing has dramatically enhanced the speedy detection of marker-trait association by eliminating the time-consuming marker detection step in the traditional BSA approach. This hybrid approach of BSA combined with sequencing was subsequently termed BSA-seq (Zhang and Panthee, 2020). BSA-seq can be regarded as a selective genotyping in which only the tails (individuals with extreme phenotypes) from a population are selected for genotyping. The tailed concept, originally proposed by Darvasi and Soller (2013), reduces the cost and simplifies the analytical process without compromising the statistical power. Rather than analyzing each individual, bulking all the individuals from each tail to create two pools significantly reduces the sequencing cost. BSA-Seq is comparatively an expeditious approach to accomplish the bulk segregant analysis by NGS. BSA conjugated with NGS ensures the rapid identification of both qualitative and quantitative trait loci (Zhang et al., 2021) and speeds up the recognition of candidate genes controlling relevant agronomic traits in diverse crop species (Liang et al., 2020). It can be applied to any population with significant phenotypic differences (Dakouri et al., 2018). For BSA-seq to be more efficient and fruitful, comparatively high sequencing depth and coverage are needed to distinguish significant SNP-trait associations. This results in a sharp rise in the sequencing cost (Zhang et al., 2021), which curbs the application of BSA-Seq to species with large genomes (Tang et al., 2018). However, BSA-seq requires only two sequencing reactions for two pools, thus compensating for high depth and coverage. Nevertheless, for an efficient and productive BSA-seq experiment, the sequencing must be performed to the deepest affordable level, rather than to construct a large pool.

## General overview of the BSA-seq technique

### Creation of bulks

**FIGURE 1:**
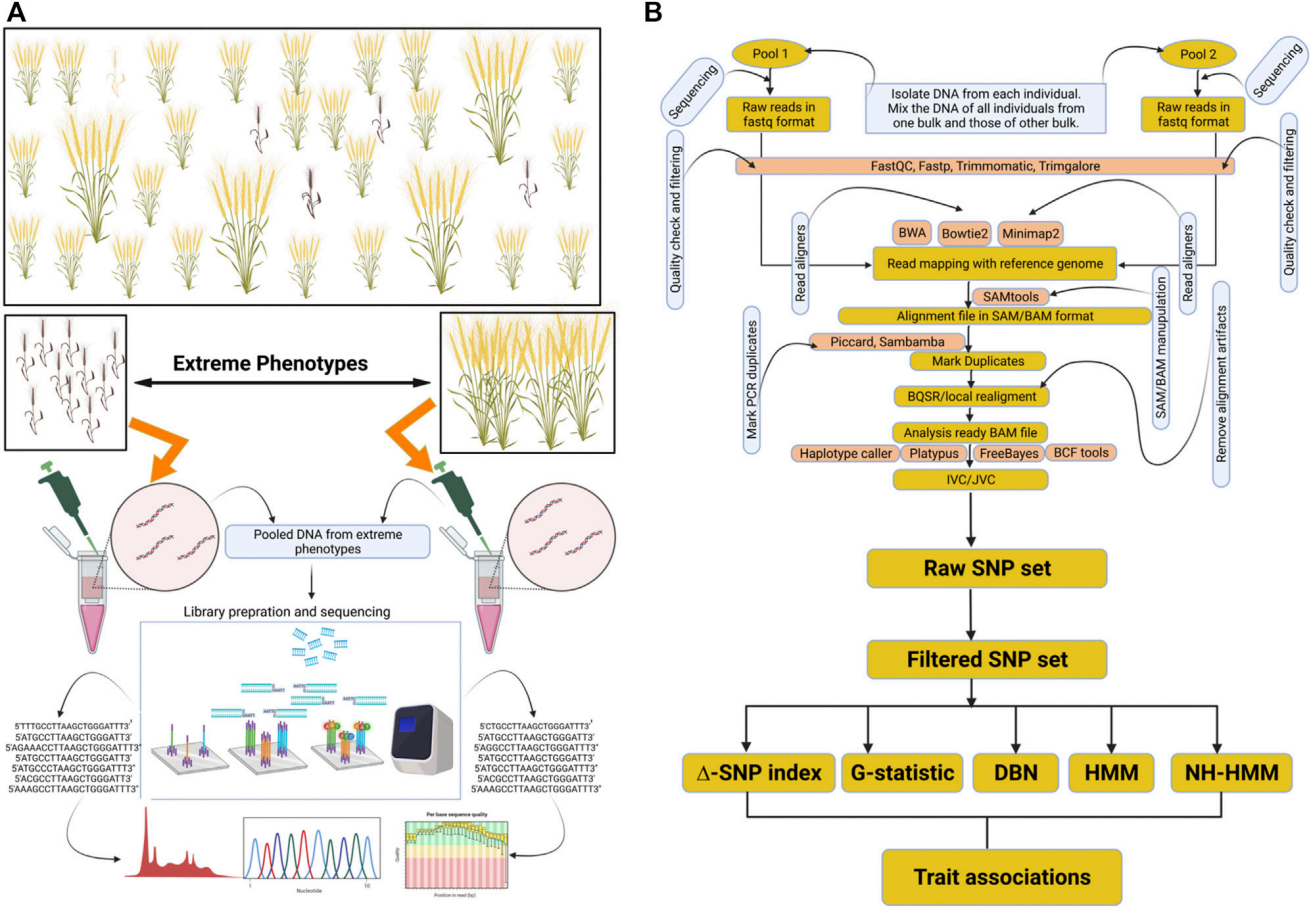
Representation of BSA-seq and general data analysis approach for marker trait associations. **(A)** depicts the creation of opposite bulks and their sequencing. **(B)** depicts variant identification and their association with the trait. This figure was created through Biorender https://biorender.com.

For the fast-track identification of QTLs linked with a particular trait of interest, a mapping population has to be constructed from a cross between parents encompassing contrasting attributes (Figure 1A). From the progeny of this cross, the individuals exhibiting contrasting phenotypes for a particular trait are initially identified. These contrasting individuals would form two different groups/bulks. For example, some individuals may be resistant to a disease, thereby forming one group/bulk, whereas the other individuals showing susceptibility to the disease form another contrasting group/bulk. Then, the DNA of the individuals from each group is extracted, and all the DNA samples of one group are pooled to create one bulk, and those of the other group are pooled separately to create a second bulk (Song et al. (2017)). After that, sequencing libraries are prepared from the pooled DNA samples of each group/bulk, followed by sequencing of the libraries using an appropriate sequencing platform (Yuexiong et al., 2020; Zhan et al., 2020).

### Sequencing and variant calling

There are diverse variant calling approaches, with no constraints for appraising a single technique, to call SNPs. Usually, the application of a variant calling technique depends on the organism and the depth and coverage of the sequencing data. The differences in the depth and breadth of sequencing coverage have implications on variant calling. Researchers have a choice to use a particular sequencing strategy, depending on their budget. The bulked samples can be sequenced by using different approaches like whole-genome sequencing, genotyping by sequencing (GBS), restriction site-associated DNA sequencing (RAD-seq), etc. The outcome of the SNP calling depends on the sequencing strategy used. Whatever the sequencing strategy used, the downstream analysis of the sequenced reads, in the fastQ/fasta format, involves aligning them with a reference genome or a *de novo* genome assembly. A standard reference genome of a species is used for this purpose; however, owing to the fact that a single reference genome could not cover all the diversities present within a species, a pangenome concept has emerged to resolve this issue. Therefore, it would be more advantageous to sequence the genome of at least one parent and use it for aligning the reads of two bulks (Luo et al., 2019; Bayer et al., 2020; Kumar et al., 2020). Read alignment represents a critical step of data analysis. Common alignment tools include BWA (Li and Durbin, 2010), Bowtie2 (Langmead and Salzberg, 2012), and Minimap2 (Li, 2018). The resulting alignments are stored and sorted in the SAM/BAM format. BAM is preferred and has become the standard format due to its compressed size and indexed nature. Manipulation of the BAM file is mostly performed through the SAMtools package (Li et al., 2009). After read alignment with the reference genome, the next step is to identify and remove the duplicated reads, i.e., the reads originating from the same genomic region. The duplicated reads may arise due to the amplification of the same fragment several times during library preparation. Picard (http://broadinstitute.github.io/picard), Sambamba (Tarasov et al., 2015), etc., are used to identify and mark these PCR duplicates in the BAM file for downstream exclusion. Before variant calling, some SNP calling pipelines utilize additional processing steps; for example, the GATK Best Practices workflow (Van der Auwera et al., 2013) performs adjustments to base quality scores of sequencing reads (base quality score recalibration (BQSR)) to remove the alignment artifacts and to reduce false positives through local realignment. However, BQSR/realignment has been found to marginally improve the variant calling accuracy; therefore, these steps may be considered optional (Koboldt, 2020). A myriad of tools have been developed for variant calling accurately like FreeBayes (Garrison and Marth, 2012), GATK HaplotypeCaller (DePristo et al., 2011), Platypus (Rimmer et al., 2014), SAMtools/BCFtools (Li, 2011), TASSEL (Bradbury et al., 2007), etc. Studies have shown that different callers produce similar results (with 90% concordance), and the differences arise only around the low coverage and low confidence regions. Despite these low differences, the differently called genome-wide variants by different callers could amount to 1,000s, necessitating the need for benchmarking and fine-tuning the variant caller (Koboldt, 2020). Choosing a single tool is usually sufficient; however, variants called through different callers can be integrated for sensitive advantage. Various tools like BCFtools are used for this purpose. Whatever the tools used, variant calling can be performed in two ways: individual variant calling (IVC) and joint variant calling (JVC). In IVC, variants are called to create a VCF file for each sample separately, followed by the merging of individual VCF files through BCFtools or other packages. One of the main problems with IVC is that since VCF files contain positional information of variants only, it is not possible to distinguish whether a variation absent in some samples is a wild type or just has low coverage to be called a variant (Koboldt, 2020). In JVC, all the samples are called simultaneously and produce genotypes at each variant position for all samples, which has the potential to resolve the above problem of the IVC. The JVC can also infer the likely genotype of a sample based on the information from the other, which provides a sensitive advantage around low coverage regions (DePristo et al., 2011; Koboldt, 2020). Errors during short-read alignment can produce artifacts during variant calling (Li, 2014). In addition, artifacts may arise due to low-quality base calls, local misalignment around indels, erroneous alignments around low complexity regions, and paralogous alignments of reads not well represented in the reference. These artifacts have been excellently described by Koboldt (2020). Such false positives usually skip during automated filtration, so a visual cross-check using genome browsers is needed to review the alignment of variants.

### Approaches for downstream data analysis

When the principle of BSA-seq for the mapping of QTLs is simple, a myriad of statistical methods have been developed to analyze BSA-seq data (Figure 1B). A more convenient and robust pipeline called PyBSASeq was developed by Zhang and Panthee (2020). Once the SNPs are generated by the variant caller, generally, the next step is to filter them based on certain criteria. The unmapped SNPs, missing SNPs, SNPs with more than one alternate (ALT) allele, and the SNPs with low-quality scores are excluded (Zhang and Panthee, 2020). The filtered SNPs are then subjected to Fischer’s exact test to obtain a set of significant SNPs. Identification of SNPs is accomplished by matching the bases from sequencing data to the reference genome. Each identified SNP is compared with the reference genome and designated as REF (reference SNP) if identical with the reference genome or ALT (alternate SNP) if not identical with the reference genome. Now, after dividing the number of ALT SNPs by the total number of SNPs (REF + ALT), an allele frequency measure termed the SNP index is obtained. The difference in SNP indices between the two bulks is termed the ∆ SNP index. For any SNP, the greater the value of its ∆ SNP index, the higher is the probability that SNP is associated with the trait of interest (Zhang and Panthee, 2020). In BSA, the alleles associated with a trait get enriched in either bulk. Therefore, if a gene contributes to a trait, its alleles and, therefore, the SNPs within that gene are enriched in either bulk. For example, in one bulk, there may be more REF allele-containing reads, whereas the other bulk may contain more ALT allele-containing reads. Due to the phenomenon of linkage disequilibrium (LD), the SNPs flanking this gene are also enriched, depending on their closeness to the gene. Based on the quantification of enrichment values of these trait-associated flanking SNPs, a recent python algorithm has been developed to analyze the BSA-seq data more simply and effectively (Zhang and Panthee, 2020). The pipeline can also calculate a G-statistic value for each SNP through the G-test using both REF and ALT SNPs in each bulk. The higher the G-statistic value, the more likely the SNP is linked with the trait. After the calculation of the ∆ SNP index or the G-statistic, a sliding window algorithm is utilized to aid the visualization. The sliding windows may contain 100s to 1000s of SNPs among which only a few can be significant. The pipeline developed by Zhang and Panthee (2020) uses the ratio of significant SNPs and the total number of SNPs within a sliding window as an indicator of the trait-associated gene within the sliding window. The greater the ratio, the higher is the probability of the sliding window containing the trait-associated gene. This approach is referred to as the significant SNP method. When using the ∆ SNP index or the G-statistic values during window sliding, the higher ∆ SNP index and G-statistic values indicate that the window under observation contains the trait-associated gene (Zhang and Panthee, 2020). Based on the plots derived from the sliding window approach, candidate genomic regions can be distinguished. The candidate genomic regions would be the windows containing the trait-associated gene. These candidate regions or QTLs associated with a particular trait can then be validated using diverse polymorphic markers (Arikit et al., 2019). Next, the SNPs within the candidate regions can be annotated using distinct bioinformatics tools. PyBSASeq is simple, effective, and highly sensitive. It performs better at low sequence coverage; therefore, it has the potential to significantly reduce the sequencing costs. It can calculate significance levels of the detected associations; however, it suffers from the deficiency of the estimation of confidence intervals for the detected QTLs. It may be resolved in future versions.

Block regression mapping (BRM) is another robust approach developed to analyze BSA-seq data comprehensively. This method was developed by Huang et al. (2020). The authors developed this algorithm to solve two key issues in analyzing BSA-seq data: 1) to accurately determine the significance threshold and 2) to determine the confidence interval of the QTLs. These two issues remain associated with QTL-seq, as claimed by Huang et al. (2020). Through the BRM approach, the users can also integrate the results from the BRM pipeline with the Pooled QTL Heritability Estimator (PQHE) (Tang et al., 2018) to estimate the heritability. The method is based on a null hypothesis (Ho), which if an allele is not associated with the trait, the frequency of that allele in two pools is equal. Conversely, the difference in frequencies of an allele between the two pools is equal to zero if the allele is not associated with the trait. However, under this condition, both the pools should be a random sample from the population for the marker. At Ho

$${\text{f}}_{1}{\;=\text{ f}}_{2}\;=\text{ f}\;\mathrm{or}\;\Delta {\text{f }=\text{ f}}_{1}{\;-\text{ f}}_{2}\;=\text{ 0}$$

where f = frequency of an allele in a population, f_1_ = frequency of an allele in pool-1, and f_2_ = frequency of an allele in pool-2. If an allele is linked with a trait, then

$$\Delta {\text{f }=\text{ f}}_{1}{\;-\text{ f}}_{2}\neq \text{0}$$

The larger the value of ∆f for an allele, the more strongly the allele is associated with the trait (Huang et al., 2020). After calculating the ∆f value for each marker, a continuous ∆f curve can be plotted across the genome. QTLs can be identified from this curve as their peaks. The local regression method LOESS (Jacoby, 2000) is used to fit the ∆f, f_1_, and f_2_ curves, followed by block regression. To calculate the significance levels, the method relies on the fact that ∆f approximately follows a normal distribution under the central limit theorem. Therefore, the significance level of the ∆f is calculated using a two-tailed test. If it is significant, the alternative hypothesis is accepted, i.e., there is QTL present in this peak. Then, the confidence interval is derived as the region between the left and right intersection points of a horizontal line (calculated mathematically) with the curve. This region represents the 95% confidence interval of the QTL (Huang et al., 2020). Bonferroni correction is used for multiple testing. Here, f1 and f2 are equivalent to SNP indices, and ∆f is the ∆SNP index of the QTL-seq method of Takagi et al. (2013a) and PyBSASeq of Zhang and Panthee (2020). The main advantage of the BRM approach is that it can calculate significance levels through multiple testing and determine the confidence intervals.

Among the other statistical approaches developed to analyze BSA-seq data, a G-statistic-based approach developed by Magwene et al. (2011) is well known. It calculates the G-statistic value for each SNP through a smoothed version of the G-test using both REF and ALT SNPs in each bulk. The higher the G-statistic value, the more likely the SNP is linked with the trait. This method takes into consideration the allele frequency variation due to bulks and variation due to sequencing of bulks. Larger bulk sizes and enough sequencing depth have the potential to detect even weak effect QTLs (Magwene et al., 2011). Although this approach is simple, Huang et al. (2020) have asserted that the method of calculating FDR for multiple testing is not concretely devised, confidence intervals cannot be estimated through this method, and it is less effective under low sequencing depth.

The MULTIPOOL method was developed by Edwards and Gifford (2012) for genetic mapping through the utilization of pooled genotyping. This approach was focused on experiments with model organisms, where the progeny of a cross is grouped and pooled based on phenotypes. Its theme is simple: a marker not linked with a trait shall segregate with equal frequency in both pools, whereas the marker linked with a trait shall be enriched in either pool. It was developed to handle larger data sets containing 1000s of markers. It uses the dynamic Bayesian network (DBN) approach for estimating confidence intervals and statistical accuracy of QTLs. The method can be used for any number of replicates and multiple experimental designs (Edwards and Gifford, 2012). It uses a probabilistic multi-locus dynamic Bayesian network model, wherein a single chromosome is considered at a time to model the influence of pool size and recombination on the frequency of neighboring alleles and describes the allele frequency change across the chromosome. Although MULTIPOOL does not rely on a specific read aligner or SNP calling strategy, however, it suffers from the problem of estimating the LOD threshold and judging the significance of signals accurately.

A simpler and widely accepted method known as QTL-seq was developed by Takagi et al. (2013b) to identify the QTLs in rice recombinant inbred lines (RIL) and F_2_ populations but can be applied to any population for detecting genomic regions that underwent artificial or natural selection. It can also be applied to populations under different environmental conditions like high and low temperatures. However, this method is not suitable for detecting minor effect QTLs as replicated measurements are not possible for each genotype. The approach uses the ∆ SNP index method. It first calculates k (number of reads having an allele different from the reference); then, the SNP index is calculated using the formula

SNP-index = k 1/n,

where n = total number of reads.

QTL-seq estimates the contribution of each parent to the variation. If SNP index = 0, there is no variation and all SNPs are the same as reference. If SNP index = 1, all SNPs belong to either parent and if SNP index = 0.5, each parent contributes equally to the variation. Generally, only the SNPs with SNP index > 0.3 in either bulk are retained for downstream analysis. A sliding window is then applied to visualize the graphs based on the SNP index. After that, the ∆ SNP index is calculated for all genomic regions, and the regions exhibiting a higher ∆ SNP index than the background genome represent the regions associated with the trait of interest. These regions correspond to peaks or the valleys of the SNP index plot (Takagi et al., 2013a). Depending on the type of genotyping, i.e., whether analysis involves allele frequencies of both tails of the phenotypic distribution of a targeted trait (bidirectional selective genotyping) (Zhang et al., 2003) or the allele frequencies from only one tail (unidirectional selective genotyping) (Foolad et al., 2001), the QTL-seq can be termed as bidirectional and unidirectional QTL-seq, respectively. Although this selective genotyping of one or both phenotypic extremes has the potential to detect effective QTLs, a simulation study evaluating the power and precision of unidirectional and bidirectional approaches revealed that the latter is more powerful than the former (Navabi et al., 2009). QTL-seq has been successfully used in a myriad of species like tomato (Illa-Berenguer et al., 2015; Wen et al., 2019), capsicum (Park et al., 2019), groundnut (Pandey et al., 2017; Clevenger et al., 2018), watermelon (Branham et al., 2018; Cho et al., 2021), bottle gourd (Chanda et al., 2018; Song et al., 2020), pear (Xue et al., 2017), radish (Hu et al., 2022), rice (Lei et al., 2020), soybean (Zhang et al., 2018), etc. QTL-seq is the most popular and widely used tool for BSA-seq analysis and has the most citations (Table 1). However, Huang et al. (2020) opine that the significance threshold estimated in QTL-seq is inappropriate, and there is no estimation of confidence interval.

**TABLE 1:** Key characteristics of different statistical approaches and pipelines used to analyze BSA-seq data.

	Method	Key statistics used	Citations	Limitation	Advantage
1	G-statistic	G-test	200	Based on estimating the G′ threshold; the method for calculating FDR for multiple testing has not been concretely devised; significantly affected by sequencing depth and is less suitable under low sequencing depth; no estimation of confidence intervals	Simplicity
2	MULTIPOOL	Probabilistic multi-locus dynamic Bayesian network model	70	Based on estimating the LOD threshold, judging the significance of signals	Non-reliance on a particular aligner and SNP calling strategy
3	QTL-Seq	SNP index, ∆ SNP index	780	Significance threshold estimated in QTL-seq is inappropriate; no estimation of confidence interval	Simplicity and intuition
4	EXPLoRA	Hidden Markov model (HMM), Linkage disequilibrium (LD)	45	No multiple testing correction, sometimes maps a single QTL as two or more adjacent QTLs, no confidence interval estimation	Robust even at a low signal-to-noise ratio
5	Hidden Markov model	HMM	9	Does not take into account that co-segregation of SNPs is affected by the distance between them	
6	Non-homogeneous hidden Markov model	HMM	16	Takes the effect of distance between SNPs during co-segregation into account	
7	QTG-Seq	smooth LOD test, Euclidean distance, and G-statistic	49	Large pool size and high sequencing coverage required	Time- and cost-saving strategy for fine-mapping, suitable for minor-effect QTLs, mapping resolution up to the gene level, and requires only four generations from the first cross of any parent lines for fine-mapping
8	PyBSASeq	Fischer’s exact test, ∆ SNP index or G-statistic, significant SNP method	4	No estimation of confidence intervals for detected QTLs	Simple and effective, calculates significance, can detect SNP-trait associations at lower sequencing coverage so can reduce up to 80% sequencing cost, high sensitivity
9	Block regression mapping	Δf or ∆ SNP index, Δf curve LOESS analysis, block regression, central limit theorem, and Bonferroni correction	10	Not apparent yet	Calculates significance, uses multiple testing, estimates confidence intervals
10	QTLseqr	∆ SNP index and G-statistic	93	Not apparent	Calculates significance, uses multiple testing, and options for better visualization

Several approaches have also been developed that rely on the hidden Markov model (HMM) concept to link SNPs with the phenotype. The HMM is used to explain or derive the probabilistic characteristic of any random process. It is used to describe the observed events that depend on hidden events. HMMs capture the hidden information from observed sequential events. In HMM, the system being modeled is assumed to be a Markov process with unknown parameters, and the observed parameters are used to determine the hidden parameters. The latter is used for further analysis (Hundal et al., 2016; Lan et al., 2017). The EXPLoRA method was developed by Duitama et al. (2014) to precisely distinguish between true and spurious linked regions for a trait of interest. This algorithm relies on linkage disequilibrium and uses HMM to model the relationships between neighboring markers. This algorithm is robust and performs better under a low signal-to-noise ratio. This tool is claimed to give better results when the true linkage signal is diluted by the availability of few segregants, sampling, and screening errors (Duitama et al., 2014). EXPLoRA is effective even at a low signal-to-noise ratio, but no multiple testing correction and confidence interval estimation are carried out. Another approach named the hidden Markov model (HMM) was developed by Calaesen and Burzykowski (2015) to analyze the BSA-seq data. The model assumes different states of a nucleotide, and each state in an offspring being same or different compared to the parent. Transition of nucleotides implies transition in states. By calculating the probabilities of transitions and states, the most probable state of each SNP is selected, which indicates the most probable genomic regions associated with the trait (Calaesen and Burzykowski, 2015). Through this method, each identified SNP is classified into one of the several predefined states having their specific biological interpretation. The HMM identified states allow the identification of genomic regions containing genes governing the trait. This method is based on the assumption that the identified SNPs are equally spaced across the whole genome, which may not always be the case. Furthermore, the co-segregation of SNPs is affected by the distance between them. Taking these two issues into consideration, an extended method of the HMM known as the non-homogeneous hidden Markov model (NH HMM) was developed by Ghavidel et al. (2015), which takes the distance between SNPs into account.

The quantitative trait gene sequencing (QTG-Seq) method was developed by Zhang et al. (2019) to accelerate QTL fine-mapping. The method partitions QTLs to convert a quantitative trait into a near-qualitative trait. The partitioning is performed by selfing the individuals heterozygous for the target QTL and homozygous for other QTLs. This is followed by mining, in which bulked pools are sequenced. In addition to the Euclidean distance and G-statistic, a new statistic called smooth LOD was used to delimit the QTL to a small interval (Zhang et al., 2019). For the determination of minor-effect QTLs and fine-mapping, QTG-Seq is cost-effective and time-saving but at the cost of a large pool size and high sequencing coverage required. The details and key features of these approaches are presented in Table 1.

## BSR-Seq approach

SNPs can be deduced from the transcriptomic data also; therefore, it is also possible to use the RNA-sequencing technology to efficiently identify SNPs from bulks. This integration of BSA and transcriptome is known as bulked segregant analysis RNA-seq (BSR-Seq). The fundamental principles of BSR-Seq would remain the same as that of the traditional BSA-seq, with the difference that only the transcribed genome is used as a data source. BSR-Seq has been applied for the elucidation of important genomic regions and SNPs associated with different traits in both plants and animals. For example, Wang et al. (2013) identified 1,255 and 56,419 differentially expressed genes (DEGs) and SNPs, respectively, between resistant and susceptible pools against enteric septicemia in catfish. By pooling the RNA samples from 12 homozygous F_3_ resistant lines to the stem rust pathogen (strain Ug99 F_3_) and 11 susceptible homozygous lines, Edae and Rouse (2019) could map the stem rust resistance to a 3.2-Mbp region on chromosome 2U of *Ae. umbellulata,* with two nucleotide-binding and leucine-rich repeat (NLR) genes as the potential candidate genes (Edae and Rouse, 2019). Moreover, BSR-Seq was used to clone the *glossy3* (*gl3*) gene of maize (Liu et al., 2012). In addition, the molecular details of wheat powdery mildew resistance through BSR-Seq revealed that a single dominant gene on chromosome 5DS conferred resistance (Zhu et al., 2020). BSR-seq was also used to identify DEGs and SNPs associated with waterlogging tolerance (Du et al., 2017). Through the BSR-seq technique, the regulatory network of melon color was identified by Chayut et al. (2015). The cold tolerance response of *Actinidia arguta* through BSR-Seq revealed that soluble sucrose and β-amylase activity were enhanced in tolerant population compared to susceptible population (Lin et al., 2021). Through BSR-Seq, in addition to the QTL regions, the differential expression of candidate genes is also achieved. However, for traits affected by the environment and the traits determined by many minor genes, BSR-Seq may not be very effective (Edae and Rouse, 2019).

## MutMap approach

With the advent of the sequencing technologies, there has been a rapid progress in deciphering the causative alleles for a particular trait more quickly than traditional QTL mapping approaches. Spontaneous mutations and activation of natural mutagens like transposons and viruses, etc., are the main sources of variation in the natural populations. If variations are not sufficient in a natural population, then artificial mutagens like EMS, acridine dyes, base analogs, and UV, X-, and gamma-rays can be used to induce mutation (Raina et al., 2016, 2020). The mutations caused by these agents lead to altered phenotypes through the generation of SNPs, indels, or segmental breaks (Tribhuvan et al., 2018). The phenotypic variations either existing spontaneously or induced artificially are exploited to map the causative genes/QTLs using an appropriate marker system. When the phenotypic variation is artificially induced to create a mutant phenotype, then the causative mutation can be analyzed and identified through MutMap methods. These methods include MutMap, MutMap+, SHOREmap (SHOrtREad map), MutMap-Gap, and NGM (next-generation mapping). First, a mutant phenotype is created through mutagenesis which is then crossed with the parent to create F_1_ and F_2_ populations. With the aid of a marker system like SNPs, the mutant phenotype is screened for segregation in the filial generations and thus mapped on the genome. The key to these approaches is the utilization of traditional BSA to generate SNP data. They exploit the power of NGS technologies to map inherited traits across any plant species where the generation of an F_2_ mapping populations is possible. SHOREmap was developed by Schneeberger et al. (2009) to identify the causative gene mutation for slow growth and pale green leaves in *Arabidopsis*. The authors first generated mutant lines and crossed it with distant parent to dilute the distribution pattern of non-causal SNPs present throughout the genome. Next, they created a bulk of 500 mutant individuals, and their DNA was pooled and sequenced. SNPs were identified between the parent and mutant. The basic idea of this technique is that among the progenies of *mutant x parent*, those with the mutant phenotype are assumed to have SNP distribution similar to mutants at the loci controlling the mutant phenotype, whereas other loci have a random distribution. NGM mapping was developed by Austin et al. (2011) to identify the cell wall biosynthesis and maintenance genes in *Arabidopsis*. NGM is similar to SHOREmap except that it utilizes less mutant lines (10) to create a bulk, without compromising the power to detect causal mutation. Like SHOREmap and NGM, the MutMap technique, which was developed by Abe et al. (2012), also uses the creation of bulks from mutant F_2_ progenies (Etherington et al., 2014). MutMap, however, differs from the aforementioned two techniques in the sense that while the latter utilizes distantly related mapping lines, the former relies on the crossing between the mutant and its wild type itself. This approach of MutMap directly targets the causal SNPs generated during mutagenesis (Tribhuvan et al., 2018). The SNPs, which are associated with the mutant phenotype, would show 0% wild type and 100% mutant reads, whereas the unlinked SNPs would show 50% each. Abe et al. (2012) developed an SNP index as the number of mutant SNP reads divided by the total number of SNP reads. If this index =1, it means that the SNP is highly linked to the mutant phenotype. This method is more likely to map recessive mutations. An advancement to MutMap known as MutMap+ was developed by Fekih et al. (2013) to tackle the lethal or sterile mutations, wherein F_2_ cannot be developed. Bulks of around 20–30 individuals for mutant and wild type are created at M_3_ generation, sequenced at ∼10x coverage followed by SNP identification in both bulks. Then, the SNP index for both is calculated, and the ∆ SNP index is derived by subtracting the wild-type SNP index from the mutant SNP index. The positive ∆ SNP index values indicate that SNP is linked with the phenotype. A further extension of MutMap, to map a causal mutation with the gaped region of the genome, was developed and named MutMap-Gap (Takagi et al., 2013a, b). Here, if an SNP with index = 1 remains undetected in the reference genome, there is a possibility that such SNPs are present within the gaped regions. Then, the unassembled reads are *de novo* assembled, and the casual SNP is identified using the *de novo* assembly. The key advantages of MutMap include 1) no need of large mapping populations, 2) no need of genetic linkage maps, 3) no need of natural variation in the population, 4) time-saving and labor-effective, and 5) direct identification of casual SNPs. Key disadvantages include 1) availability of a reference genome, 2) artificial mutagenesis required to develop mutant lines, 3) maintenance of mutant lines, and 4) not applicable if a phenotype cannot be scored (Tribhuvan et al., 2018).

## Successful application of BSA-Seq in elucidation of trait-associated QTLs

A myriad of studies on diverse species have proven the applicability of BSA-seq in mapping QTLs for different traits of agronomic importance. The details of some important studies on important crop plants are presented in Table 2. BSA-Seq successfully identified the genomic regions controlling the locule number and fruit weight in tomatoes (Illa-Berenguer et al., 2015) that may lead to significant breakthroughs in fruit development in tomatoes. Breeding heat-tolerant cultivars of tomatoes seems appealing. In order to identify the heat stress-responsive QTLs in tomato, Wen et al. (2019) used an integrated approach of conventional QTL mapping, BSA-Seq, and RNA-Seq and found five major QTLs determining the trait of interest. Their results have significance in breeding for improved thermotolerance in tomatoes. A major QTL on chromosome 1 regulating capsaicinoid biosynthesis in the pericarp of capsicum was identified by Park et al. (2019) using BSA-Seq in integration with RNA-Seq. Significant yield losses and deteriorated fodder quality in groundnut are caused due to rust and late leaf spot fungal diseases. To address this issue, Pandey et al. (2017) identified three QTL loci for rust resistance and one for late leaf spot resistance using the BSA-Seq approach. Furthermore, Clevenger et al. (2018) also mapped late leaf spot resistance QTLs in groundnut by BSA-Seq. Identification of these genomic regions controlling rust and late leaf spot resistance and their introgression into elite groundnut cultivars would bring revolution in groundnut breeding. In the case of watermelon, *Fusarium* wilt, caused by the fungus *Fusarium oxysporum* f. sp. niveum (Fon) leads to significant losses in yield. Using BSA-seq coupled to QTL mapping, the genomic region controlling resistance to this fungus was fine-mapped to around the 315-kb region (Branham et al., 2018). In order to map loci determining semi-dwarfism in watermelon, Cho et al. (2021) identified a single recessive gene through BSA-Seq. Watermelons are severely affected by heat stress. Grafting watermelon to heat-tolerant bottle gourd rootstocks is one solution to this problem (Yang Y. et al., 2013a). So understanding of the inheritance and identification of loci controlling heat tolerance in bottle gourd may lead to significant breakthroughs in watermelon breeding for heat tolerance. Using BSA-Seq, seven heat-tolerant QTLs with one as a major effect QTL for heat tolerance in bottle gourd were determined (Song et al., 2020). In addition, bottle gourd rootstock is used to improve cold tolerance and disease resistance in cucurbits. The aphid-transmitted papaya ringspot virus watermelon strain (PRSV-W) and zucchini yellow mosaic virus (ZYMV) are the two most common viruses infecting bottle gourd. Through BSA-seq, fine-mapping of the *Prs* locus and identification of the candidate resistance gene for PRSV-W were elucidated (Chanda et al., 2018). The red peel of pears is more attractive and also provides health benefits to consumers. So genes controlling the skin coloration aid in cultivar selection and enhance pear breeding. Using BSA-seq, a 582.5-kb candidate genomic region associated with red/green skin (R/G) locus, harboring approximately 81 predicted protein-coding genes, was identified (Xue et al., 2017). Further fine-mapping and elucidation of the specific casual genes would enhance the commercial value of pears. An important commercial attribute of radish is its root shape, measured as the ratio of the root length to root diameter. Hu et al. (2022) identified seven QTLs distributed on five chromosomes controlling the root shape. The results of this study are significant for fine-mapping and functional analysis of root-shaped QTLs and cultivar breeding for the root shape in radish (Hu et al., 2022). Among the abiotic stresses, salt stress negatively affects all crop species, leading to compromised plant performance and significant losses in crop yield. Seedling survival and overall yield in rice are directly affected by salt tolerance at the bud burst stage. Using BSA-seq, a major candidate region on chromosome 7 was identified, which was further narrowed down to a 222-kb genomic interval. Furthermore, five differentially expressed genes (DEGs) were identified in this candidate region through the RNA-seq approach at the bud burst stage under the salt-treated condition. In addition, the expression of one gene, (*OsSAP16*), was enhanced under drought stress, implying that *OsSAP16* is the strong candidate gene (Lei et al., 2020). These results are significant for improving the salt tolerance of rice varieties. Grain size and weight are important traits that determine the overall yield in cereal crops. In order to identify the candidate genomic regions controlling the grain size and weight in rice, Yaobin et al. (2018 used BSA-seq and identified a 15–20 Mb region on chromosome 5. Plant height is closely related to soybean yield. Using QTL-seq, Zhang et al. (2018) identified a 1.73-Mb region on chromosome 13. Linkage mapping was used to confirm this region in the mapping population. Candidate gene analysis revealed that *Glyma.13 g249400* showed significantly higher expression in soybean plants with greater plant height; therefore, it can be a strong candidate gene for this trait (Zhang et al. (2018).

**TABLE 2:** Details of the studies utilizing BSA-Seq for the elucidation of trait-specific genomic regions in different crop species.

S.No.	Species	Pop type	Pop size	Pool size	Sequencing strategy	Number of SNPs	Bioinformatics approach used	Trait	Key findings	Reference
1	Rice	NIL-F_2_	176	35 individuals from extreme phenotypes	Whole-genome sequencing (WGS)	455,262	QTL-seq method	Grain length and weight	One major QTL, 15–20 Mb on chr 5, for grain length and weight identified	Yaobin et al. (2018)
2	Rice	F_3_	10,800	385 tolerant pools and 430 sensitive pools	Paired-end Illumina sequencing on Hiseq 2000 platform	450,000	G′ statistic method	Cold tolerance	Six QTLs were mapped on chromosomes 1, 2, 5, 8, and 10	Yang et al. (2013b)
3	Rice	F_2_	940	20 extreme phenotypes for heading time (HT) and plant height (PH)	WGS on the Illumina HiSeq X Ten platform	511,393 for HT and 543,319 for PH	ΔSNP-index method	Heading time and plant height	Four QTLs for HT on chromosomes 3, 6, 9 and 10. Three QTLs for PH on chromosomes 1 and 8	Zhang et al. (2021)
4	Rice	RILs	190	20 extreme phenotypes were used for bulking	Paired-end sequencing using the Illumina MiSeq platform	184,917	Euclidean distance and ΔSNP-index method	Cold tolerance	One major QTL on chr6 was identified, which spans 1.81 Mb and harbors 269 genes	Sun et al. (2018)
5	Rice	RILs	151	----------	Paired-end sequencing using Illumina HiSeq 2500	116,993	Euclidean distance and ΔSNP-index method	Grain shape	One major QTL on chr9 was identified, which spans 0.8 Mb and harbors 101 genes	Wu et al. (2020)
6	Cucumber	F_2_	258	10 individuals from extreme phenotypes	Illumina paired-end sequencing	234,393	Δ (SNP index)	Early flowering	One major QTL around 890 kb on chr 1 for early flowering. The gene Csa1G651710 was identified as the main flowering switch	Lu et al. (2014)
7	Cucumber	F_3_	135	15 resistant and 15 susceptible	Paired-end sequencing using Illumina HiSeq 2000	933,846 and 915,524 for susceptible and resistant bulk	ΔSNP-index method	Vein yellowing virus resistance	A unique region in chromosome 5 containing 24 annotated genes was identified for resistance	Pujol et al. (2019)
8	Maize	RILs	224	46 more extreme plants formed two pools	Paired-end sequencing using Illumina HiSeq 2000	3,301,371	Customized R-script	Flowering time and plant height	Two major QTLs found for FT on chr 5 and chr 8 were 10.8 Mb and 18.9 Mb in size, respectively. Two major QTLs on chr 4 and chr 6 found for PH were 21.2 Mb and 9.7 Mb in size, respectively	Haase et al. (2015)
9	Maize	ILs	400	10 tolerant and 10 sensitive extreme phenotypes	BSR-seq	114,580	Bayes’ theorem	Waterlogging	In tolerant and sensitive bulks, 354 and 1,094 genes were differentially expressed, respectively. GRMZM2G055704 on chromosome 1 was identified as a candidate gene responsive to waterlogging	Du et al. (2017)
10	Wheat	RILs	244	six low- and six high-TGW	SLAF-seq	132,530	ΔSNP-index method	1,000 grain weight (TGW)	One candidate gene associated with TGW was identified on chr 7A	Hu et al. (2016)
11	Hessian fly (HF), a wheat galling parasite	Non-structured Louisiana field population	---	23 virulent and 19 avirulent	WGS	1.5 million	Fisher’s exact test using PoPoolation2	Hessian fly (HF) virulence to wheat R genes H6, Hdic, and H5	One 1.3-Mb region for HF virulence was mapped to HF autosome 2	Navarro-Escalante et al. (2020)
12	Chickpea	F_4_	221	10 individuals of each low and high seed weight forming two pools	Paired-end WGS using the Illumina HiSeq 2000 platform	118,321	Δ (SNP index)	100 seed weight	One major QTL of 35 kb on chromosome 1 containing six genes	Das et al. (2015)
13	Chickpea	RILs	92 and 139 for two populations	10 and 14 extreme phenotypes for two populations	WGS	77,938 in one population and 106,907 in the other	G-statistic and ΔSNP-index method	Ascochyta blight resistance	17 QTLs identified and mapped on chromosomes Ca1, Ca2, Ca4, Ca6, and Ca7	Deokar et al. (2019)
14	Tomato	F_2_ populations	549	10 individuals of each extreme phenotype	Paired-end WGS using the Illumina HiSeq 2000 platform	----------	Δ (SNP index)	Fruit weight (FW) and locule number (LC)	Three highly significant and newly mapped FW QTLs on chr 1 and chr 11. 66 candidate genes for FW. Three LC QTLs of low significance	Illa-Berenguer et al. (2015)
15	Groundnut	RILs	266	25 individuals with extreme phenotypes	Paired-end WGS using Illumina HiSeq 2000	259,621 for rust and 243,262 for LS	Δ (SNP index)	Rust and late leaf spot disease	One 3.06-Mb region on the A03 pseudomolecule of A-genome harboring 3,136 SNPs was identified for rust resistance. A 2.98 Mb region on A03 pseudomolecule harboring 66 SNPs was identified for LS resistance	Pandey et al. (2017)
1F6	Groundnut	RILs	366	20 individuals with extreme phenotypes	WGS	10,759	ΔSNP-index method	Fresh seed dormancy	Two genomic regions on the B05 and A09 pseudomolecules control seed dormancy	Kumar et al. (2020)
17	Potato	Diploid mapping population	90	10 individuals with extreme phenotypes	Paired-end Illumina HiSeq 2000	6,766,8,152,000	Pearson’s chi-squared test	Steroidal glycoalkaloids (GAs)	One region located on chromosome 1 ranging from 63.1 to 73.5 Mb was found the most confident	Kaminski et al. (2016)
18	Pepper	F_2_	249	30 individuals with extreme phenotypes	SLAF-seq	106,848	Euclidean distance	first flower node	One QTL on chr 12 was detected, followed by 393 high-quality SNP markers associated with FFN	Zhang et al. (2018)
19	Sunflower	F_2_ and F_3_	300	15 individuals with extreme phenotypes	Genotyping-by-sequencing (GBS)	11,484	G-statistic using QTLseqr	Broomrape resistance	Two major QTLs on chromosome 3	Imerovski et al. (2019)
20	Rapeseed	BC_8_	965	36 individuals with extreme phenotypes	Illumina HiSeq 2000 platform	1,830,225	ΔSNP-index method	Plant architecture	Five major QTLs on chromosome 1	Ye et al. (2022)
21	Pigeonpea	F_2_	179	15 individuals with extreme phenotypes	WGS	47,429, 46,510, and 54,556 for three different bulk types	ΔSNP-index method	Days to flowering (DTF)	Two significant genomic regions, one on CcLG03 and another on CcLG08 were found controlling DTF	Singh et al. (2022)

In addition to its importance in crop plants, BSA-seq is also widely used in other species as well like yeast (Wenger et al., 2010; Hu et al., 2015), *Tilapia* fishes (Gu et al., 2018), etc. Using MULTIPOOL, Vogel et al. (2021) was able to dissect the genomic regions controlling the root and crown rot resistance against phytophthora in squash fish, whereas De Witt et al. (2019) and Wang et al. (2019) were successful in elucidating the unique alleles involved in lignocellulosic inhibitor tolerance and genomic variants linked to high-temperature fermentation performance in yeast, respectively. Furthermore, Trindade de Carvalho et al. (2017) successfully used BSA-seq in yeast through EXPLoRA that relies on the hidden Markov model (HMM).

## Potential of BSA-seq in medicinal plant genomics

Medicinal plants are less explored at the genomic level as compared to staple crop plants. Even the breeding programs for the genetic improvement of medicinal plants are at the pioneering stage, and the development of trait-specific homogeneous lines is far away from reality. However, BSA-seq can greatly speed up and facilitate their breeding programs by making the use of F_2_ generations. Thus, medicinal plant breeders can get a general idea about the nature of the progenies in the context of a specific trait by integrating BSA with NGS technologies. This information can then be used to develop trait-specific homogeneous lines through selfing of the selected lines. Practically, this approach is feasible for only those medicinal plants that have less generation time and flower early in life. Some examples of such medicinal plants that are best suited for which reference genome is available and hence BSA-seq can serve their breeding purpose effectively may include stevia, tea, and tulsi. For longer generation time in medicinal plants, especially tree species, the creation of F_2_ generation is almost impossible.

## Conclusion and future prospects

BSA-seq and its related approaches have the potential to quickly identify the trait-specific genomic regions/QTLs in a high-throughput manner. It takes the advantage of traditional BSA in integration with rapidly evolving NGS technologies. The most admirable attribute of this approach is that it takes only F_2_ generations to precisely identify trait-specific genomic regions/QTLs, thereby saving much time. However, this is achieved at the cost of additional capital investment for deep sequencing. Therefore, there is a trade-off between time and capital investment in using BSA-seq. With the rapid advancement of NGS technologies and a steep decrease in the cost of sequencing, it is expected that in near future, the sequencing depth would not be a matter of concern while estimating the overall cost of BSA-seq.
